# Methylation analysis of the DAPK1 gene in imatinib-resistant chronic myeloid leukemia patients

**DOI:** 10.3892/ol.2014.2677

**Published:** 2014-11-06

**Authors:** SELCEN CELIK, DILARA AKCORA, TULIN OZKAN, NURAY VAROL, SENA AYDOS, ASUMAN SUNGUROGLU

**Affiliations:** 1 Department of Basic Biotechnology, Institute of Biotechnology, Ankara University, Golbasi, Ankara 06830, Turkey; 2 Department of Medical Biology, Faculty of Medicine, Ankara University, Sihhiye, Ankara 06100, Turkey; 3 Department of Biology, Faculty of Arts and Sciences, Mehmet Akif Ersoy University, Burdur 15100, Turkey

**Keywords:** DAPK1, DNA methylation, chronic myeloid leukemia, imatinib resistance, methylation-specific polymerase chain reaction

## Abstract

Death-associated protein kinase-1 (*DAPK1*) is a pro-apoptotic gene that induces cellular apoptosis in response to internal and external apoptotic stimulants. The silencing of *DAPK1* can result in uncontrolled cell proliferation, indicating that it may have a role in tumor suppression. *DAPK1* activity can be inhibited by the cytosine methylation that occurs in its promoter region. These methylation changes in the promoter region of *DAPK1* have been reported in a range of solid and hematological malignancies. In the present study, *DAPK1* methylation was investigated in chronic myeloid leukemia patients (n=43) using bisulfite conversion followed by methylation-specific polymerase chain reaction. The present study included a number of patients who were identified to be resistant to the common chemotherapeutic agent imatinib (STI571, Gleevec^®^, Glivec^®^), exhibiting at least one mutation in the breakpoint cluster region-Abelson murine leukemia (*BCR-ABL*) gene. Thus, the patients in the present study were divided into two groups according to their response to imatinib therapy: Non-resistant (n=26) and resistant (n=17) to imatinib. Resistant patients were characterized by the presence of single or multiple mutations of the *BCR-ABL* gene: i) T315I, ii) M351T, iii) E255K, iv) T315I and M351T or v) T315I, M351T and E255K. The present study identified that: i) The incidence of *DAPK1* methylation was significantly higher in the resistant patients compared with the non-resistant patients; ii) the extent of resistance varied between mutation types; and iii) there was no *DAPK1* methylation in any of the healthy controls. These findings indicate that *DAPK1* methylation may be associated with a signaling pathway for imatinib resistance in chronic myeloid leukemia.

## Introduction

The death-associated protein kinase-1 (*DAPK1*) gene is localized to chromosome 9q34.1 and encodes a 160-kDa serine/threonine, microfilament-bound kinase which is involved in interferon-γ, tumor necrosis factor-α and Fas ligand-induced apoptosis, anoikis and autophagic cell death, respectively (1–4). The DAPK1 protein has a kinase domain, a calmodulin regulatory domain, ankirin repeats, P-loops, a microfilament-binding domain and a death domain (5), allowing it to be involved in various signaling pathways within the cell. For example, DAPK1 interacts with extracellular signal-regulated kinase 1/2 (ERK1/2) via its death domain. This interaction causes ERK to induce DAPK1 phosphorylation at Ser 735, which enhances the catalytic activity of DAPK1. DAPK1 activity contributes to the arrest of ERK in the cytoplasm, thus, blocking cell proliferation regulated by the RAS/RAF/ERK signaling pathway. This reciprocal relationship between DAPK1 and ERK may be involved in the regulation of apoptosis (6).

DNA methylation occurs within CpG dinucleotides of the mammalian genome. DNA methyltransferase enzymes (DNMTs) catalyze the addition of a methyl group (−CH_3_) to the 5′ position of cytosine, resulting in methylated cytosine, termed 5-methylcytosine (5meC) (7). DNA methylation is hypothesized to be involved in transcriptional silencing (8), and loss of DNA methylation appears to be associated with cellular differentiation (9–11) and cancer growth (12–14). Specific agents, such as 5-azacytidine and 5-aza-2′-deoxycytidine (decitabine), inhibit DNA methylation by blocking DNMT activity (15,16) and have been proposed for use in cancer therapy (15,17–19).

*DAPK1* methylation may be associated with the loss of DAPK1 activity, as increased methylation in the *DAPK1* promoter region has been detected in various types of cancer, such as renal (20) and cervical cancers (21), B cell lymphoma (22), myelodysplastic syndrome, acute myeloblastic leukemia (23) and chronic myeloid leukemia (CML) (24–26). CML is a myeloproliferative disorder resulting from the oncogenic transformation of hematopoietic stem cells, and is characterized by the Philadelphia chromosome, a reciprocal translocation between the exon 2 sequence upstream of the Abelson murine leukemia (*ABL*) proto-oncogene on chromosome 9 and the 5′ sequence of the breakpoint cluster region (*BCR*) gene on chromosome 22. The transcript of oncogenic *BCR-ABL* is a 210-kDa protein with tyrosine kinase activity that is present in the cytoplasm and activates mitogenic and anti-apoptotic pathways (27,28). Imatinib (STI571, Gleevec^®^, Glivec^®^) is typically used to inhibit the tyrosine kinase activity of the BCR-ABL protein in CML therapy; however, specific CML patients are unresponsive to imatinib treatment (29). Mutations within *BCR-ABL* cause increased BCR-ABL expression levels and, therefore, these patients consequently develop imatinib resistance. These mutations include T315I (in the imatinib-binding domain of BCR-ABL), M351T (in the catalytic domain) and E255K (in the ATP-binding domain) (30,31).

The present study aimed to investigate whether *DAPK1* methylation occurs in CML patients with or without imatinib resistance, and identified that: i) The *DAPK1* promoter was significantly methylated in CML patients (10/43) compared with healthy individuals (0/25); ii) the proportion of imatinib-resistant CML patients demonstrating *DAPK1* methylation (6/17) was higher than the proportion of non-resistant CML patients demonstrating *DAPK1* methylation (4/26); and iii) the incidence of *DAPK1* methylation in resistant patients varied between the different types of *BCR-ABL* mutation. The results of the present study indicate that *DAPK1* methylation may be associated with resistance to imatinib therapy in CML patients; however, this is dependent on the type of mutation causing the resistance.

## Materials and methods

### Samples

Blood samples were obtained from 43 CML adults who had enrolled in clinical assessment for imatinib therapy. The samples were screened for resistance to imatinib and for the presence of *DAPK1* methylation. Additionally, control blood samples were collected from 25 healthy adults. All participants were enrolled at the Department of Medical Biology, Faculty of Medicine, Ankara University (Ankara, Turkey). Informed consent was obtained from all participants and the research protocol was approved by Ankara No. 1 Clinical Research Ethics Committee (Ankara, Turkey).

### DNA isolation

DNA samples from peripheral blood were isolated using the salt precipitation method. Briefly, the cells were lysed on ice for 1 h in 1.54 M lysis buffer*,* followed by incubation with 1X sodium chloride-tris-EDTA and 10% SDS (Fisher Scientific, Pittsburgh, PA, USA), and incubated with 0.865 M proteinase K (Sigma, St. Louis, MO, USA) at 37°C overnight. Whole blood cells were subsequently treated with 5.6 M NaCl and centrifuged at 750 × g for 20 min, and the resultant DNA samples were incubated overnight in distilled water at 37°C.

### BCR-ABL mutation analysis

Mutations conferring imatinib resistance were detected using allele-specific oligonucleotide polymerase chain reaction (ASO-PCR). PCR reactions for T315I, M351T (33) and E255K (33) were performed as previously described. The primer sequences for T315I were as follows: Forward, 5′-GCC CCC CTT CTA TAT CAT CAC-3′ for normal PCR; forward, 5′-GCC CCC CTT CTA TAT CAT CAT-3′ for ASO-PCR; and reverse, 5′-GGA TGA AGT TTT TCT TCT CCA-3′. The primer sequences for M351T were as follows: Forward, 5′-CCA CTC AGA TCT CGT CAG CCA T-3′ for normal PCR; forward, 5′-CCA CTC AGA TCT CGT CAG CCA C-3′ for ASO-PCR; and reverse, 5′-GCC CTG AGA CCT CCT AGG CT-3′. The primer sequences for E255K were as follows: Forward, 5′-GCG GGG GCC AGT ACG GGG-3′ for normal PCR; forward, 5′-GCG GGG GCC AGT ACG GGA-3′ for ASO-PCR; and reverse, 5′-GCC AAT GAA GCC CTC GGA C-3′. The predicted PCR products are 158, 149 and 192 bp for T315I, M351T and E255K, respectively.

### Sodium bisulfite modification of DNA

A CpGenome™ DNA Modification kit (cat. no. S7820; EMD Millipore, Billerica, MA, USA) was used to modify the DNA, according to the manufacturer’s instructions. Briefly, this included reagent preparation, DNA modification, initial desalting, the completion of DNA modification (desulfonation), second desalting and elution.

### Methylation-specific PCR (MS-PCR)

**Figure 1:**
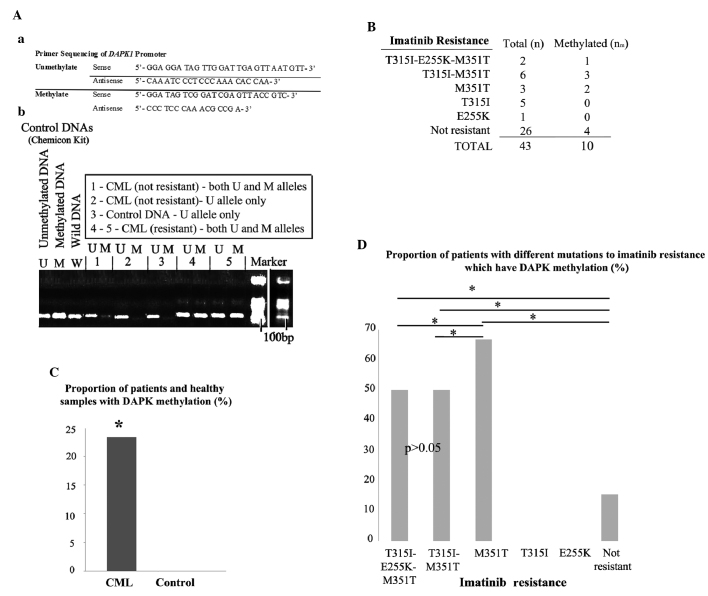
Analysis of *DAPK1* methylation. (Aa) *DAPK p*rimer sequences used for methylation analysis performed using methylation-specific PCR following bisulfite conversion. (Ab) Representative PCR products for U and M alleles of *DAPK1* in resistant (samples 4 and 5) and non-resistant (samples 1 and 2) patients, in a control sample from healthy individuals (sample 3), as well as in control U, M and W DNA samples. (B) The total number of samples and the number of samples exhibiting *DAPK1* methylation. (C) The proportion (%) of samples with *DAPK1* methylation is significantly greater in CML patients compared with the healthy controls. (D) The proportion (%) of patients with DAPK1 methylation varied among different mutations. The majority of patients with M351T demonstrated *DAPK1* methylation; however, none of patients with T315I or E255K were methylated, and no difference was detected between the patients with triple (T315I-M351T-E255K) and double (T315I-M351T) mutations (P>0.05). Furthermore, a relatively low number of non-resistant patients demonstrated *DAPK1* methylation (P<0.05). ^*^P<0.05. *DAPK1*, death-associated protein kinase-1; PCR, polymerase chain reaction; U, unmethylated; M, methylated; W, wild type; CML, chronic myeloid leukemia.

A CpG WIZ^®^ DAP-Kinase Amplification kit (cat. no. S7801; EMD Millipore) was used to amplify the DNA. The primer sequences designed for the *DAPK1* promoter are indicated in Fig. 1Aa. The amplification kit included unmethylated (U), methylated (M) and wild-type (W) DNA. Prior to performing PCR, the U and M DNAs were sodium bisulfite-modified. MS-PCR was conducted by performing 40 cycles of 95°C for 5 min, 95°C for 45 sec, 58°C for 45 sec and 72°C for 60 sec, and the PCR products for the M and U alleles were finally defined by performing 2% agarose gel electrophoresis. The predicted PCR products are 105, 97 and 99 bp for U, M and W DNA, respectively.

### Statistical analysis

Statistical analysis was performed using SPSS software (version 17.0; SPSS, Inc., Chicago, IL, USA) and graphs were constructed using SPSS or Microsoft Excel software (Microsoft Corporation, Redmond, WA, USA). The proportion (%) of samples demonstrating *DAPK1* methylation was arcsine-transformed and compared using a Mann-Whitney U test. P<0.05 was considered to indicate a statistically significant difference.

## Results

### Determination of imatinib resistance mutations

**Figure 2:**
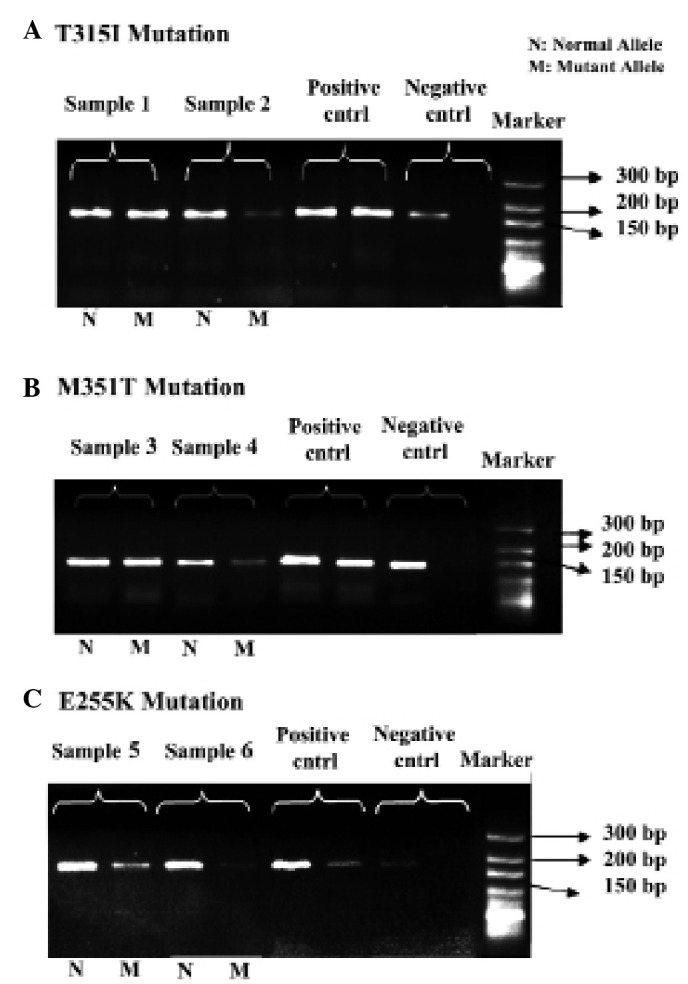
Analysis of breakpoint cluster region-Abelson murine leukemia mutations. Imatinib resistance in chronic myeloid leukemia patients was detected using allele-specific oligonucleotide PCR. Representative PCR products are indicated for the (A) T315I, (B) M351T and (C) E255K mutations. Mutant samples have a normal and mutant allele, but normal samples only have a normal allele. PCR, polymerase chain reaction; Positive cntrl, samples from patients known to be resistant to imatinib treatment; Negative ctrl, samples from healthy individuals; Cntrl, control.

Initially, the mutation profiles for the imatinib-resistant CML patients (determining the existence of T315I, M351T and E255K mutations) were examined using ASO-PCR. The cohort was assembled from numerous CML patients who had experienced failed therapies at various stages of the disease and, therefore, had applied to The Department of Medical Biology, Faculty of Medicine, Ankara University (Ankara, Turkey) for investigation into imatinib unresponsiveness. As predicted, these patients were determined to have at least one of the resistance mutations examined and, as such, were described as resistant to imatinib (Fig. 2A–C). The patients who were clinically responsive to imatinib were determined to have normal *BCR-ABL* alleles and were described as non-resistant to imatinib. Imatinib resistance was detected regardless of the stage of CML and, therefore, patients were divided into just two groups: Resistant and non-resistant.

### Methylation analysis of the DAPK1 gene

Subsequently, the existence of methylation in the promoter region of the *DAPK1* gene was determined using sodium bisulfite modification of the DNA samples, followed by MS-PCR. The primer sequences designed for the U and M alleles of the *DAPK1* promoter region are demonstrated in Fig. 1Aa, and representative PCR products for the U and M alleles of the *DAPK1* gene are demonstrated in Fig. 1Ab. The patients with a U allele alone were described as not methylated (i.e., samples 2 and 3) and the patients exhibiting an additional M allele (i.e., samples 1, 4 and 5; Fig. 1Ab) were described as methylated. As expected, none of the healthy individuals (control; n=0/25) exhibited a methylated *DAPK1* promoter region; however, almost 25% of CML patients (resistant and non-resistant) exhibited *DAPK1* methylation (P<0.05; Fig. 1B). The proportion of patients with or without *DAPK1* methylation are provided in Fig. 1C. No methylation was detected in patients with T315I (0/5) or E255K (0/1), and 4/26 non-resistant patients demonstrated *DAPK1* methylation (Fig. 1C). A detailed comparison of the proportion (%) of patients with *DAPK1* methylation between the different BCR-ABL mutation groups is indicated in Fig. 1D. Compared with the other mutation groups, the highest proportion of *DAPK1* methylation was detected in the M351T alone mutation group (P<0.05) and the lowest proportion was observed in the non-resistant patients (P<0.05; Fig. 1D). However, no significant difference was identified in the proportion (%) of patients with *DAPK1* methylation between those with triple (T315I, E255K and M351T) and double (T315I and M351T) mutations (P>0.05; Fig. 1D). Furthermore, no methylation was detected in patients exhibiting T315I or E255K alone (Fig. 1D).

## Discussion

The DAPK1 protein is known to be involved in the suppression of cancer formation and metastasis via apoptosis and, thus, is considered to be a tumor suppressor gene (1). CpG methylation in the *DAPK1* promoter region has been detected in a range of solid cancers, such as non-small cell lung cancer (34), leiomyosarcoma (35), nasopharyngeal carcinoma (36), and hematological malignancies, such as follicular lymphoma (37) and CML (24,25,38). A previous study determined that 50% of CML patients exhibited *DAPK1* methylation, and this was not correlated with age, hematological parameters, chromosomal abnormalities or the type and quantity of the *BCR*/*ABL* transcripts, however, it was correlated with gender and CML phase (26). In intestinal system cancers, *DAPK1* activity was inhibited by a protein complex that included DNMT1. Furthermore, deacetylation of histones H3 and H4 appeared to contribute to *DAPK1* silencing (39). These factors indicate that the silencing of *DAPK1* by such methylation may be associated with cancer progression. To the best of our knowledge, however, no study has been conducted investigating the correlation between the presence of methylation in the *DAPK1* promoter region and mutations that confer resistance to imatinib therapy in CML patients. The present study examined whether *DAPK1* methylation occurred in CML patients with or without resistance to imatinib. DNA methylation (resulting in 5meC) is typically detected using bisulfite sequencing. This methodology is based on the discrimination between methylated and unmethylated cytosines by treatment with sodium bisulfite followed by MS-PCR. However, with the discovery of novel 5meC modifications [for example, 5hmC (5-hydroxymethylcytosine)] (40–42), it has been demonstrated that standard sodium bisulfite treatment is unable to distinguish between 5meC and 5hmC (43,44). Following bisulfite treatment, 5hmC is converted to cytosine-5-methylensulfonate (CMS) and CMS is read as 5meC (43). The development of an additional DNA treatment is therefore required for the accurate discrimination of 5meC from other DNA modifications. Various attempts to improve this discrimination with additional steps have been conducted, for example, ten-eleven translocation methylcytosine dioxygenase (TET)-assisted bisulfite sequencing, which includes glucosylation and TET oxidation of genomic DNA, resulting in the discrimination of 5meC from 5hmC (45,46); the conversion of 5hmC to CMS to provide genome scale profile of 5hmC (47); and chemical modification of 5-carboxylcytosine (5caC) to provide base resolution detection of 5caC (48). Gold standard bisulfite-based methodologies using a standard sodium bisulfite treatment are able to reveal an overall profile of 5meC modifications, rather than a 5meC profile alone, although caution is required when interpreting the changes in DNA methylation as they do not reflect the changes in the individual 5meC metabolites. The present study identified that the *DAPK1* gene is significantly methylated (including possible hydroxymethylation) in CML patients and this is correlated with mutations that result in resistance to imatinib. Notably, one in two of the patients exhibiting both T315I and M351T mutations demonstrated DNA methylation in the *DAPK1* promoter region compared with non-resistant patients; however, none of the patients with T315I alone demonstrated *DAPK1* methylation. Patients exhibiting the E255K mutation alone or with other mutations were not identified to have *DAPK1* methylation. Furthermore, it was observed that the PCR product bands of the M alleles for the *DAPK1* promoter were thicker in the resistant CML patients compared with the non-resistant patients (and a similar variation was observed in the PCR products of the various resistance mutations). Although the band thickness may indicate the difference in the level of methylation of the *DAPK1* gene, the present study did not explore the extent of methylation but instead aimed to identify whether methylation exists in the *DAPK1* promoter region. The results of the present study may indicate that methylation of the *DAPK1* promoter region is associated with a specific signaling pathway(s) in the resistance to imatinib.

Imatinib resistance can be induced by point mutations (such as T315I and M351T) of transgenic *BCR-ABL* and this results in increased expression of the oncogenic BCR-ABL fusion protein. BCR-ABL may be involved in various cell proliferation pathways within the cells, such as rat sarcoma (RAS), Janus kinase/signal transducer and activator of transcription, phosphatidylinositol 3-kinase and Myc (1,49). The protein complex of BCR-ABL with growth factor receptor bound protein 2 and son of sevenless activates the RAS pathway (49), and RAS activates mitogen-activated protein kinase (MAPK) and MEK1/2 (a MAPK kinase), resulting in the transportation of extracellular signal-regulated kinase (ERK) from the cytosol to the nucleus (1). Furthermore, DAPK1 induces apoptosis when it is phosphorylated by the RAS-ERK signaling pathway (50). The death domain of the DAPK1 protein interacts with ERK (6) and ERK is arrested in the cytoplasm. Thus, the increase in the expression level of the *BCR-ABL* transcript may not be eliminated if the *DAPK1*-regulated apoptosis pathway is inhibited. The results of the present study indicate that *DAPK1* methylation may be involved in imatinib resistance in CML depending on the type of BCR-ABL mutation; however, *DAPK1* may not have a direct effect on the suppression of overexpressed tyrosine kinase in CML patients. Furthermore, it is possible that imatinib resistance may not depend on BCR-ABL activity (51); therefore, the proposed contribution of DAPK1 to the BCR-ABL pathway requires a detailed investigation. An additional study may be required to investigate whether imatinib resistance induces *DAPK1* methylation or vice versa.

Regarding the possible involvement of DNA methylation in CML*,* the additional use of demethylating agents (such as decitabine) may be useful in CML treatment. The level of global methylation (as assessed by the methylation of the retrotransposable element of the human genome, long interspersed element-1 gene) was relatively greater in patients responding to a combined treatment of imatinib with decitabine compared with non-responder patients (18,19); furthermore, patients without imatinib resistance demonstrated a higher rate of response to this combination therapy (19). This indicates that patients with imatinib resistance may be less responsive to the combined use of decitabine with imatinib. However, the additional use of a demethylating agent with imatinib may induce apoptosis in CML, as this combined treatment inhibited imatinib resistant cell growth *in vitro* to some extent compared with decitabine treatment alone (52). Therefore, the detection of the methylation status of tumor suppressor genes (such as those involved in apoptosis) may be important for the selection of additional treatment with imatinib in CML therapy. The current problem with the use of demethylating agents is that they induce global rather than gene-specific demethylation, potentially resulting in proto-oncogene activation by demethylation (53). Demethylation of the *DAPK1* gene can be induced by a demethylating agent, allowing imatinib-resistance to be overcome. This may be useful for identifying whether *DAPK1* demethylation induced by such demethylating agents reduces the incidence of BCR-ABL mutation.

CML patients with T315I and E255K mutations were detected to be insensitive to clinically achievable doses of imatinib (54), indicating that additional agents may be required for the treatment of these patients. However, higher doses of imatinib were useful in cases of M351T and Y253F mutation (54). Therefore, the strategy for individual or combined treatment should depend on the type of mutations causing resistance. The findings of the present study indicate that the E255K mutation is not involved in the association between imatinib resistance and *DAPK1* methylation, instead, M351T is the major mutation in this association. M351T (which occurs in the catalytic domain of BCR-ABL) may be more emphasized than mutations in other domains as it increases the catalytic function of kinases. However, it should be noted that some other mutations in *BCR-ABL* that were not examined in the present study (such as Y253H, F317L and H396R) (31) require investigation to achieve a broader comparison.

In conclusion, the current study presents a typical trend for the association between the presence of *DAPK1* promoter methylation and a variety of *BCR-ABL* mutations, regardless of any characteristics of the CML patients, such tumor stage, gender or age. The present study provides an insight into the understanding of imatinib resistance in CML progression and proposes that the determination of *DAPK1* methylation may be a criterion to use an additional agent in the treatment of CML (i.e., decitabine). Furthermore, the methylation status of various other tumor suppressor genes may be useful for the determination of an accurate CML treatment strategy. This is important as cancer is a complex disease formed by the genetic and epigenetic mechanisms of multiple genes.
